# Regulation of membrane excitability: a convergence on voltage-gated sodium conductance

**DOI:** 10.1007/s12035-014-8674-0

**Published:** 2014-03-29

**Authors:** Wei-Hsiang Lin, Richard A. Baines

**Affiliations:** Faculty of Life Sciences, University of Manchester, Oxford Road, Manchester, UK

**Keywords:** Excitability, *Drosophila*, Epilepsy, *Paralytic*, Splicing, Translational repression

## Abstract

The voltage-gated sodium channel (Na_v_) plays a key role in regulation of neuronal excitability. Aberrant regulation of Na_v_ expression and/or function can result in an imbalance in neuronal activity which can progress to epilepsy. Regulation of Na_v_ activity is achieved by coordination of a multitude of mechanisms including RNA alternative splicing and translational repression. Understanding of these regulatory mechanisms is complicated by extensive genetic redundancy: the mammalian genome encodes ten Na_v_s. By contrast, the genome of the fruitfly, *Drosophila melanogaster*, contains just one Na_v_ homologue, encoded by *paralytic* (*DmNa*_*v*_). Analysis of splicing in *DmNa*_*v*_ shows variants exhibit distinct gating properties including varying magnitudes of persistent sodium current (I_NaP_). Splicing by Pasilla, an identified RNA splicing factor, alters I_NaP_ magnitude as part of an activity-dependent mechanism. Enhanced I_NaP_ promotes membrane hyperexcitability that is associated with seizure-like behaviour in *Drosophila*. Nova-2, a mammalian Pasilla homologue, has also been linked to splicing of Na_v_s and, moreover, mouse gene knockouts display seizure-like behaviour.

Expression level of Na_v_s is also regulated through a mechanism of translational repression in both flies and mammals. The translational repressor Pumilio (Pum) can bind to *Na*_*v*_ transcripts and repress the normal process of translation, thus regulating sodium current (I_Na_) density in neurons. Pum2-deficient mice exhibit spontaneous EEG abnormalities. Taken together, aberrant regulation of Na_v_ function and/or expression is often epileptogenic. As such, a better understanding of regulation of membrane excitability through RNA alternative splicing and translational repression of Na_v_s should provide new leads to treat epilepsy.

## Introduction

The regulation of neuronal excitability—primarily the ability to maintain action potential firing within physiological constraints—is an important mechanism for maintenance of neuronal stability [1]. Without such regulation, chronic changes in levels of synaptic excitation have the potential to destabilise neural circuits leading to an imbalance in neuronal activity. One consequence of activity imbalance is seizure, which if recurrent is termed epilepsy [2]. The voltage-gated sodium channel (Na_v_) plays a key role in the regulation of neuronal excitability because its activation results in action potential firing. It is perhaps, therefore, not surprising that many mechanisms that act to stabilise neuronal activity do so through modifying the activity of this class of ion channel [1, 3–7].

Ten genes (*SCN1A-SCN11A*), encoding pore-forming *α*-subunits, are present in mammals [8]. This relatively high number is, however, insufficient to support the wide diversity of Na_v_ kinetics reported in the nervous system. Diversity of signalling is critically reliant on additional mechanisms such as alternative splicing, RNA editing, and protein modification (i.e., phosphorylation) [9–12, 4]. However, whilst the importance of posttranscriptional and posttranslational modifications is appreciated for refining activity of channel subtypes, understanding of the mechanisms that neurons employ to determine which form of Na_v_ to express remains rudimentary. In contrast to mammals, the genome of the fruitfly *Drosophila melanogaster* contains only one Na_v_ channel homologue: encoded by *paralytic* (*DmNa*_*v*_) [13, 5]. The lack of redundancy, coupled with a high degree of structural and functional homology, makes DmNa_v_ an advantageous model with which to study the role of this ion channel family [14, 15]. In this review, we use DmNa_v_ as a model to summarise recent findings relating to how neurons generate diversity in Na_v_ channel activity and to stabilise neuronal circuit function when faced with changing levels of synaptic excitation.

### Alternative splicing generates diversity in Na_v_ channel activity

Alternative splicing involves the substitution, removal, and/or inclusion of exonic sequences within a pre-messenger RNA (mRNA) to produce transcripts encoding related protein isoforms [9]. Estimates indicate that ~95 % of human genes are alternatively spliced [16, 17]. Variant transcripts of *DmNa*_*v*_, first reported by Loughney et al., (1989), were among the first evidence for the existence of alternative splicing of this family of gene products. Subsequent studies in *Drosophila*, *Musca*, and cockroach have identified 15 alternatively spliced exons [18, 19, 14, 20, 15]. Importantly, alternative splicing of exons is replicated in mammalian Na_v_ channels [21–23]. Spliced exons are conserved across evolutionarily diverged species, strongly indicative of fundamental physiological importance.

**Fig. 1:**
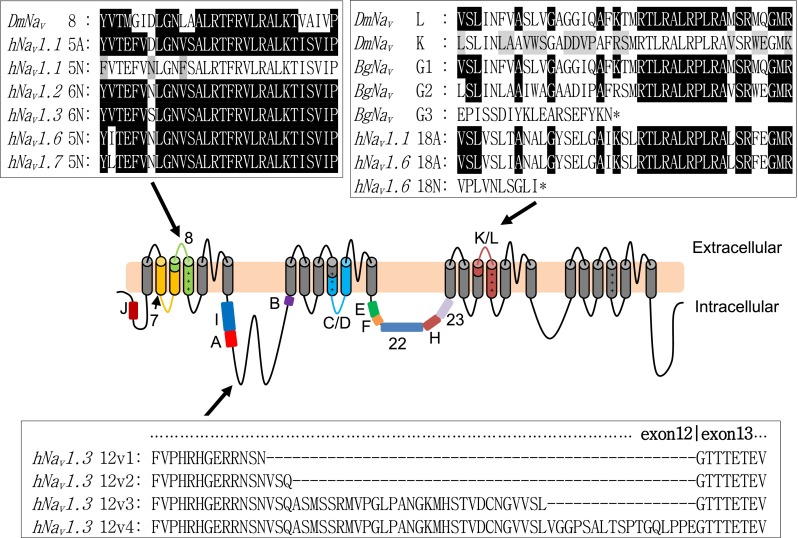
Schematic of the predicted topology of the voltage-gated sodium channel showing approximate locations of *Drosophila* spliced exons. Cytoplasmic *DmNa*_*v*_ exons *J*, *7*, *8*, *I*, *A*, *B*, *E*, *F*, *22*, *H*, *23* are optional, while exons *C*/*D* and *K*/*L* are mutually exclusive. *DmNa*_*v*_ exon 8 is conserved in human Na_v_s as mutually exclusive spliced exons 5A and 5N (6A and 6N in *hNa*_*v*_ *1.2* and *hNa*_*v*_ *1.3* due to different exon numbering in the consensus gene sequence), and identical residues are shown in *black boxes*. Exon 5A and 5N of *hNa*_*v*_ *1.1* differ by 3 amino acids, shown in *grey boxes* in the 5N sequence. Mutually exclusive *DmNa*_*v*_ spliced exon L and cockroach *BgNa*_*v*_ exon G1 are identical and are conserved in human: exon 18A of *hNa*_*v*_ *1.1* and *hNa*_*v*_ *1.6. DmNa*_*v*_ exons K and L differ by 16/41 residues (shown in *grey boxes* in the exon K sequence). Inclusion of *BgNa*_*v*_ exon G3 and *hNa*_*v*_ *1.6* exon 18N generated a truncated channel. Exon 12 of *hNa*_*v*_ *1.3* is located in the intracellular loop between domains I and II. By using different splice donor sites in *exon 12*, four spliced variants, 12v1, 12v2, 12v3, and 12v4 can be generated. The amino acid sequences are obtained as follows: *DmNa*_*v*_ 8, K, and L [15]; *hNa*_*v*_ 5A and 5N [61]; *hNa*_*v*_ *1.6* 18A and 18N [23]; *BgNa*_*v*_ G1, G2, and G3 [20]; *hNa*_*v*_ *1.3* 12v1, 12v2, 12v3, and 12v4 [30]

A recent structure-function study has described the effects to DmNa_v_ channel kinetics of alternative splicing [15]. Of the 15 known splice decisions, two splice events are mutually exclusive incorporating one of either a pair of exons (*C*/*D* and *K*/*L*). Both exon pairs are membrane spanning, contributing to domains IIS4–5 and IIIS3–4, respectively. The remaining 11 spliced exons (*J*, *7*, *8*, *I*, *A*, *B*, *E*, *F*, *22*, *H*, *23*) are independent and cytoplasmic. Heterologous expression of *DmNa*_*v*_ splice variants in *Xenopus* oocytes shows that such splicing imparts specific attributes to channel kinetics. For example, inclusion of exon *F* results in a hyperpolarising shift in activation kinetics, indicative of increased excitability for those neurons that express *F*-containing variants. By contrast, inclusion of exons *J* and *E* results in a depolarising shift of activation voltages which are predicted to reduce neuron excitability. On the other hand, channels expressing exon *H* inactivate at more depolarised voltages, predicted to make neurons more excitable. Finally, the choice to include mutually exclusive exons *K* or *L* markedly affects the magnitude of the persistent current (I_NaP_) that arises from incomplete inactivation of the channel [24, 5, 25]. Inclusion of exon *K* results in a smaller I_NaP_ relative to that observed from expression of transcripts that contain exon *L*, in otherwise identical channels. Increasing I_NaP_ leads to an increased frequency of action potential firing [26, 5]. Figure 1 summarises the known splicing events of *DmNa*_*v*_, and the effect on channel kinetics and/or I_NaP_ is summarised in Table 1. Of course, the caveat to heterologous expression is that the nature of the cell membrane of the cell type used may influence the kinetics of expressed channels compared to expression, in this instance, in *Drosophila* neurons [27]. Attempts to express *DmNa*_*v*_ variants in *Drosophila* neurons, using the well-characterised GAL4/UAS system, has repeatedly failed to produce functional channels, for unknown reasons (Lin and Baines, unpublished observations).

**Table 1:** Summary of spliced *Na*_*v*_ exons that are known to affect channel kinetics. Predicted influence on neuron excitability due to splicing are stated, increased (↑), decreased (↓), or complex (?)

Channel	Exon	Expression system	Predicted effect on cell excitability (by changing)	References
*DmNa*_*v*_	*J*, *E*	*Xenopus* oocytes	↓ (act →)	[15]
*DmNa*_*v*_	*F*	*Xenopus* oocytes	↑ (act ←)	[15]
*DmNa*_*v*_	*H*	*Xenopus* oocytes	↑ (inact →)	[15]
*DmNa*_*v*_	*K*	*Xenopus* oocytes	↓ (I_NaP_ amplitude ↓)	[15]
*DmNa*_*v*_	*L*	*Xenopus* oocytes	↑ (I_NaP_ amplitude ↑)	[15]
*hNa*_*v*_ *1.1*	*5A*	HEK293T	↑ (I_NaP_ amplitude ↑, inact →)	[61]
*hNa*_*v*_ *1.1*	*5N*	HEK293T	↓ (I_NaP_ amplitude ↓, inact ←)	[61]
*hNa*_*v*_ *1.3*	*12v1*	*Xenopus* oocytes	↓ (inact ←)	[30]
*hNa*_*v*_ *1.3*	*12v2*	*Xenopus* oocytes	↓ (act →)	[30]
*hNa*_*v*_ *1.3*	*12v3*	*Xenopus* oocytes	↑ (inact →)	[30]
*hNa*_*v*_ *1.3*	*12v4*	*Xenopus* oocytes	↑ (act ←)	[30]
*BgNa*_*v*_	*B*	*Xenopus* oocytes	↑ (I_NaT_ amplitude ↑)	[28]
*BgNa*_*v*_	*G1*	*Xenopus* oocytes	? (I_NaT_ amplitude ↓, act ←, inact →)	[20]
*BgNa*_*v*_	*G2*	*Xenopus* oocytes	? (I_NaT_ amplitude ↑, act →, inact ←)	[20]

Specific changes observed to channel kinetics are as follows: depolarising (→) or hyperpolarising (←) shifts in activation (act) or inactivation (inact) and/or increased (↑) or decreased (↓) transient (I_NaT_) or persistent sodium current (I_NaP_) amplitude

Both embryonic and adult *Drosophila* CNS expresses a wide diversity of *DmNa*_*v*_ splice forms. However, the profile of splicing differs between these two stages. This is indicative that different Na_v_ properties are required at each stage and that these differences are achieved through splicing. Differences of spliced exons expressed in these two stages include a greater usage of exon *J* (89 %) but not of exon *F* (10 %) in adults and vice versa in embryos (10 % exon *J* and 78 % exon *F*) [14, 15]. However, the physiological significance of these differences is not clear. It is interesting to note that *DmNa*_*v*_ transcripts which lack a majority of common cytoplasmically located spliced exons result in channels with shifted activation and inactivation kinetics towards hyperpolarised and depolarised voltages, respectively, and which also exhibit a much larger I_NaP_. These properties are predicted to make neurons highly excitable [15]. Similarly, analysis of splicing of *Na*_*v*_ in other insects shows that it is important for functional properties of the expressed channel. For example, exclusion of optional exon *B* (located at the linker between the domains I and II, but not equivalent to exon *B* in *Drosophila*) in cockroach sodium channels (*BgNa*_*v*_) potentiates the amplitude of the fast-activating and inactivating I_Na_ transient current (I_NaT_), which is likely to increase cell excitability (Table 1) [28]. Indeed, an emerging theme is that splicing in of optional exons primarily reduces channel activity and hence, membrane excitability, in order to suit the requirements of neural signalling.

Splicing in intracellular coding regions of mammalian Na_v_s can also result in changes to channel activity. For example, the human *Na*_*v*_ *1.3* (*SCN3A*) alternative spliced exon *12*, which encodes an intracellular loop between domains I and II, results in the generation of multiple isoforms. By using multiple splice donor sites in exon *12*, four different variants are produced: 12v1, 12v2, 12v3, and 12v4. The variant 12v4, when compared to 12v2, seemingly increases membrane excitability by shifting activation kinetics of the expressed I_Na_. By contrast, inactivation kinetics showed a shift toward hyperpolarising potentials for 12v1 over 12v3, indicative that expressing 12v1 might be expected to decrease membrane excitability (Table 1) [29, 30]. Taken together, Na_v_ gating properties can be determined by the inclusion of exons to alter membrane excitability. However, details of how inclusion of specific spliced exons change gating of the affected channels remains to be determined. A possible mechanism for altering channel kinetics is the phosphorylation state of the channel [30, 31]. Analysis of the amino acid sequence of human Na_v_1.3 splice variants revealed the presence of two additional phosphorylation sites (protein kinase C on Ser^631⁄632^ and casein kinase II on Ser^646^) in 12v3 and 12v4 that are absent from other variants [30]. Changing membrane excitability through phosphorylation in the I-II linker of Na_v_ may influence current amplitude without significantly affecting gating properties [11, 32–36].

### Persistent Na current and membrane excitability

The persistent Na current (I_NaP_) has been identified to play critical roles in regulating membrane excitability [37]. Moreover, numerous point mutations in human *Na*_*v*_s, identified in patients with epilepsy, potentiate this component of the voltage-gated Na current (I_Na_) [26]. Interestingly, I_NaP_ is also a primary target of some clinically used antiepileptic drugs, including phenytoin, valproic acid, and lamotrigine. [38–40]. It is significant, therefore, that the magnitude of this current can be altered through alternative splicing. However, our understanding of the molecular machinery that regulates splicing of *Na*_*v*_s is poor. This is unfortunate because a fuller understanding may offer new leads for antiepileptic drug design.

**Fig. 2:**
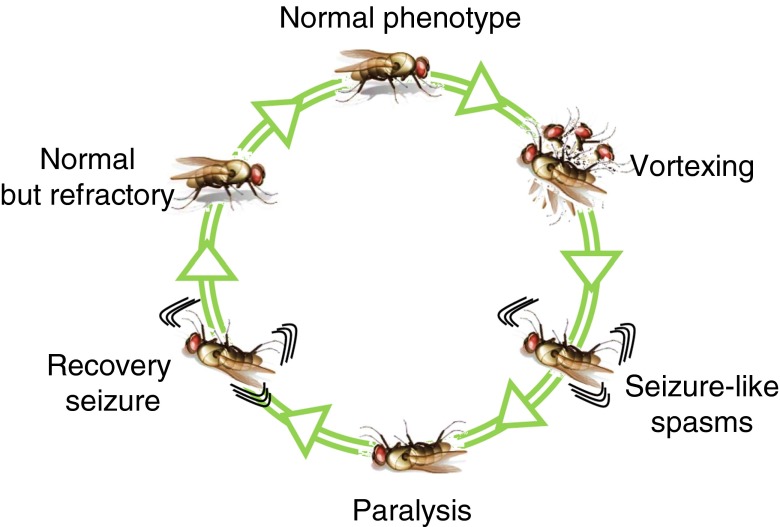
*Drosophila* bang-sensitive mutant behaviour. Brief vortexing (~10 s) of the culture vial, containing bang-sensitive mutant flies, induces a stereotyped sequence of seizure-like spasms, followed by a period of paralysis, and then a recovery seizure-like phase that precedes a normal but refractory phase followed ultimately by a complete recovery

In early behavioural screens of *Drosophila*, different single-gene mutations were identified that induce a seizure-like phenotype when flies are exposed to strong sensory stimuli. Following a mechanical shock, such as vortexing or harsh-tapping of the culture vial, bang-sensitive (bs) mutant flies exhibit a stereotyped sequence of seizure-like spasms, followed by a period of paralysis, and then a second recovery seizure-like phase that precedes a more complete recovery (Fig. 2) [41, 42]. Despite the evolutionary distance, the resemblance in epileptiform activity between fly and humans and the response to clinical antiepileptic drugs make bs mutants an accepted model for studying epilepsy [42–49]. One such bs mutant—*slamdance* (*sda*)—which has a deficiency of aminopeptidase N, exhibits increased seizure-like activity in response to electrical stimulation in the larval stage. Detailed electrophysiology shows that I_NaP_ is significantly increased in central motoneurons in this mutant [48]. A molecular analysis reveals that splicing of *DmNa*_*v*_ is similarly altered in the *sda* mutation to favour inclusion of exon *L* at the expense of exon *K* [25]. As previously described (see above), inclusion of exon *L* results in channels that, when expressed in *Xenopus* oocytes, exhibit a larger I_NaP_ [15]. Seizure-like behaviour, in response to electric shock, along with the increased I_NaP_ and increased inclusion of *L* isoform are all reversed by feeding larvae with antiepileptic drugs (AEDs) including phenytoin (Phy) and gabapentin (Gbp) [25, 48]. Thus, a better understanding of how I_NaP_ is regulated, particularly through splicing, may be beneficial for epilepsy therapy.

### Activity-dependent alternative splicing regulation of I_NaP_ expression

Seizures can be induced in both mammals and flies through ingestion of proconvulsants such as picrotoxin (PTx) [50, 46]. PTx elicits seizure through antagonism of the GABA_A_ receptor-suppressing synaptic inhibition [51, 52]. Remarkably, we showed that enhancement of synaptic activity in wild-type larvae, through ingestion of PTx, is sufficient to increase inclusion of exon *L* in *DmNa*_*v*_, increasing I_NaP_ as a consequence and inducing a bang-sensitive phenotype. Conversely, seizure activity can be rescued via enhancing synaptic inhibition in *sda* through ingestion of GABA [25]. Both manipulations suggest that the ‘decision’ to splice either exon *L* or *K* is dictated by neuronal activity: i.e. activity-dependent. Increasing synaptic excitatory input results in greater inclusion of exon *L,* which in turn increases I_NaP_ and membrane excitability [25]. Increased excitability would be predicted to further increase inclusion of exon *L* up to a maximum of 100 % (which is observed in *sda* and other bs mutants). Such a self-reinforcing cycle provides a plausible, although untested, explanation of the clinical observation in which untreated seizures beget seizures, i.e. paroxysmal activity has the potential to promote susceptibility to further seizures [53, 54].

Splicing of *Na*_*v*_ transcripts in response to activity provides an important mechanism for inducing changes in excitability. Because of the complexity of the mammalian CNS, with its larger number of expressed Na_v_s, the extent of splicing and its functional consequences are not well understood. However, the high degree of homology between DmNa_v_ and its mammalian counterparts allows us to use the former to guide future studies in mammals. *Drosophila* exon K/L (located in homology domain III S3-4) is conserved in the homologous domain III from insect to mammal [20, 18, 23], although the outcome of splicing differs. Splicing at this location in cockroach produces three mutually exclusive transcripts that contain spliced exons *G1*, *G2*, or G3. *G3* contains a stop codon and generates a nonfunctional channel, whereas G1 and G2 result in channels that differ in peak I_NaT_ amplitude, gating properties (Table 1) and sensitivity to deltamethrin, a pyrethroid insecticide [20]. In mammals, this same region is also spliced in *Na*_*v*_ *1.1* and *Na*_*v*_ *1.6*—resulting in the inclusion of exons *18A* or *18N* [21–23]. Exon *18A* predominates in adult brain and *18N* in embryo and nonneuronal tissues. Similar to the cockroach exon *G3*, mammalian exon *18N* contains a stop codon and generates a truncated channel. These truncated Na_v_ channels that contain only the first two domains, express mainly in nonneuronal tissues, and are hypothesised to be a ‘fail-safe’ mechanism to prevent the expression of functional Na_v_s in nonexcitable cells [55, 23, 21, 22].

A second splicing event in mammalian Na_v_s is noteworthy because it occurs at the equivalent S3-4 region of homologous domain I. Similar to *DmNa*_*v*_, splicing at exon *5* in *Na*_*v*_ *1.1* is mutually exclusive with the choice of either exons *5A* or *5N* (again for adult and neonatal). Alternative splicing in this region is also observed in *Na*_*v*_ *1.2*, *1.3*, *1.6* and *Na*_*v*_ *1.7* in both human and mouse [56–60]. In human Na_v_1.1, three amino acids differ between exon 5A and 5N; however, the channels exhibit distinct gating properties. Heterologous expression of human Na_v_1.1-5N, in HEK293T cells, produces channels which exhibit more rapid inactivation and reduced I_NaP_ compared to Na_v_1.1-5A. Whilst much needs to be learnt about this splice event, these results suggest that splicing at this location is sufficient to confer changes in neuronal excitability (Table 1) [61]. Intriguingly, inclusion of neonatal *exon 6N* is increased in both *Na*_*v*_ *1.2* and *Na*_*v*_ *1.3* following electrical or kainate-induced seizure in adult rat hippocampus [62, 63], perhaps indicative that splicing may similarly be activity-regulated in mammals, as it is in the fly.

### Pasilla/Nova, critical factors involved in activity-dependent alternative splicing

**Fig. 3:**
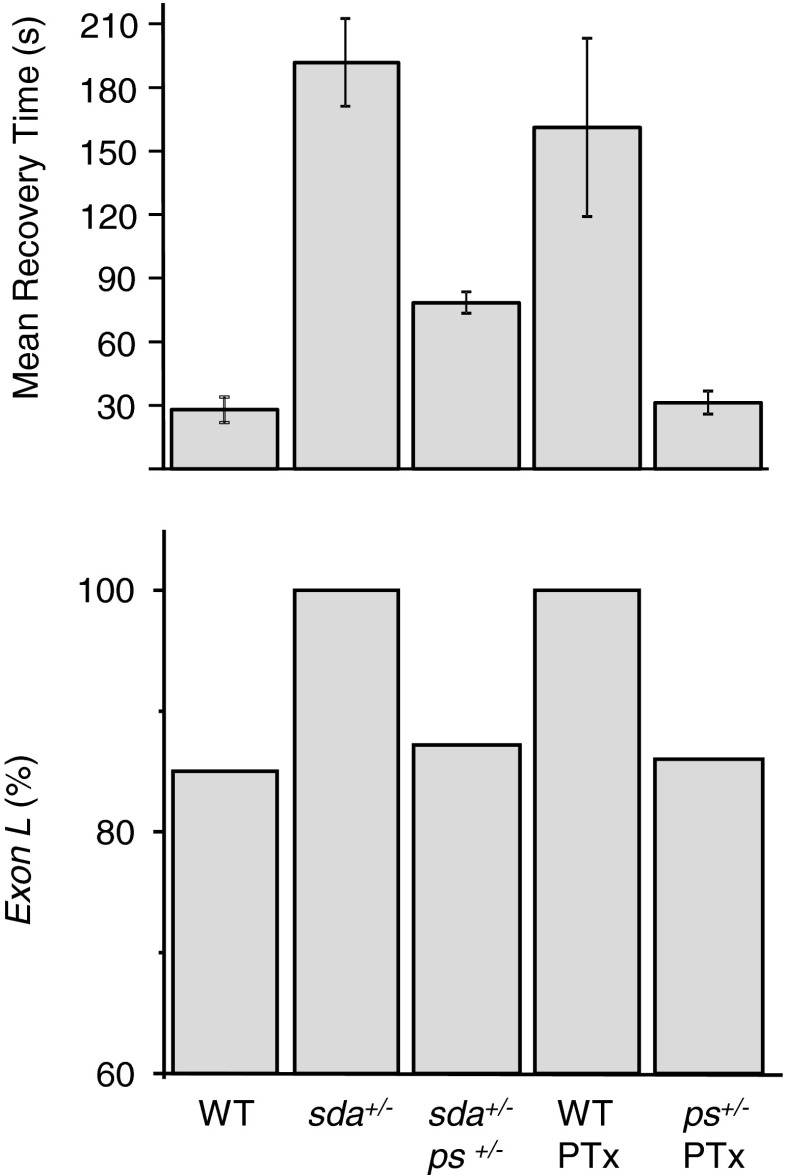
Pasilla is required for activity-dependent inclusion of exon *L* of *DmNa*_*v*_. Prolonged mean recovery time to electroshock of third instar larvae (i.e. increased severity of seizure) is observed in both slamdance (*sda)* mutants and picrotoxin (PTx)-fed WT flies. Analysis of splicing of *DmNa*_*v*_ in whole CNS of such larvae shows that inclusion of exon *L* increased to ~100 %. In *sda*, loss of one copy of *pasilla* (*sda* ^+/−^, *ps* ^+/−^) is sufficient to decrease the inclusion of exon *L* and to rescue seizure-like behaviour. Similarly, removal of one copy of *ps* in WT larvae (*ps* ^+/−^) diminishes PTx-induced seizure, as well as inclusion of exon *L*. Data are taken from [25]

A screen of RNA-binding proteins in *Drosophila* first identified Pasilla (Ps) to be sufficient to regulate splicing of mutually exclusive exons *K* and *L* in *DmNa*_*v*_ [64]. The inclusion of exon *K* is significantly increased to 50 % in a *ps* loss-of-function mutant indicating that the presence of Ps is necessary for the inclusion of exon *L* [15]. Loss of one copy of *ps* is also sufficient to rescue the bs-associated seizure behaviour of *sda* mutants and, moreover, to also prevent PTx-induced seizure in WT background (Fig. 3) [25]. These data suggest that Ps is required for the underlying activity-dependent splicing mechanism. Ps, which contains a K-homology (KH) RNA-binding domain [65, 66], encodes the *Drosophila* homologue of the human neuro-oncological ventral antigen 1 and 2 (Nova-1 and Nova-2, respectively) proteins [67, 68]. Nova-1 and Nova-2 are expressed to high levels in brain, however, in largely nonoverlapping patterns [69–71]. By recognising YCAY-motifs, located either in introns or 3’UTRs of target transcripts, Nova1/2 regulate neuronal alternative splicing and also mediate transportation of some target transcripts between the nucleus and cytoplasm [68, 72, 73]. Splicing in at least 17 ion channel genes, including *Na*_*v*_ *s*, is predicated to be regulated by Nova-2 [74, 75]. Significantly, overexpression of Nova-2 in HEK293 cells results in an increase in the *Na*_*v*_ *1.1-5N* splice variant [75]. In support of this, *Nova-2* and *Na*_*v*_ *1.1-5N* transcript abundance are upregulated in temporal neocortical tissue of mesial temporal lobe epilepsy patients. [75]. The relationship between Nova expression and epilepsy has been further examined by EEG recordings in *Nova-2* ^*+/−*^ heterozygous mice (*Nova-2* ^*−/−*^ mice die within 2–3 weeks of birth). Perturbing Nova steady-state levels in *Nova-2* ^*+/−*^ heterozygous mice gives rise to cortical hyperexcitability and also to spontaneous generalised seizure discharge [73]. Moreover, Nova localization shifts from primarily nuclear to cytoplasmic within 2–4 h after pilocarpine-induced seizure [73]. Taken together, these findings strongly implicate perturbation of Nova-2 function contributes to epileptogenesis. The corollary would be that manipulation of Nova activity might be antiepileptic. The conservation of function between Ps and Nova offers the exciting opportunity to utilise *Drosophila* to rapidly identify molecules that might influence Nova function.

### Sodium channel expression and homeostasis

Control of neuron excitability is known to be achieved at the genomic level through transcriptional regulation of *Na*_*v*_ channel genes [1, 3, 76, 6]. In *Drosophila* CNS, the regulation of the voltage-gated sodium current (I_Na_) can be achieved through activity-dependent alteration of *DmNa*_*v*_ mRNA level [77, 4, 5, 78]. Removal of excitatory synaptic inputs to motoneurons, achieved by expressing tetanus toxin light chain in all central neurons, significantly increased *DmNa*_*v*_ transcript abundance and also the magnitude of I_Na_ in motoneurons. On the other hand, enhanced excitatory synaptic release, achieved by increasing cAMP level in the CNS, decreased both mRNA level and I_Na_ [77, 5]. This homeostatic mechanism is ideally suited to allow membrane excitability to track the degree of synaptic excitation to which a neuron is exposed (i.e. neuronal homeostasis).

Mammalian neurons (e.g. rat) exhibit the same type of activity-dependent homeostasis of membrane excitability [3]. Deprivation of synaptic excitation in cortical neuron cultures, achieved by chronically blocking glutamatergic signalling, resulted in increased *Na*_*v*_ *1.6* mRNA expression, I_Na_, and membrane excitability [79]. The underling mechanism of this homeostatic regulation, in both flies and mammals, requires the protein Pumilio (Pum) [79, 80, 5, 78]. Pumilio is a member of the Pum and FBF (PuF) RNA-binding protein family [81, 82] and is evolutionarily conserved in many species including yeast (*Saccharomyces cerevisiae*), *C. elegans*, *Drosophila*, *Anopheles*, zebrafish, *Xenopus*, mouse, and human [82, 83].

In the fly CNS, activity-dependent increase in Pum level results in the translational repression of *DmNa*_*v*_ transcripts, reducing I_Na_ and membrane excitability [5, 78]. This mechanism is dynamic, such that decreasing levels of synaptic excitation results in decreased Pum level, increased *DmNa*_*v*_ transcript abundance, and potentiation of membrane excitability. In rat cortical neurons, the level of Pum was similarly observed to be activity-dependent, mirroring the mechanism observed in the fly [79, 84]. Pum is able to repress translation through binding a specific motif—termed Nanos response element (NRE) [85]—present in both *DmNa*_*v*_ and rat *Na*_*v*_ *1.6* transcripts [79, 78]. Once Pum is bound to a transcript, cofactors Nanos [86] and brain tumour [86] are recruited to form a quaternary RNA-protein complex that causes transcript deadenylation [87] and consequently repression of translation. An 8-nucleotide core motif UGUA(A/U/C)AUA [88] of the NRE is sufficient for the binding of Pum to *DmNa*_*v*_ transcripts [78], and this motif exists in about 10 % of all *Drosophila* transcripts [88]. Notably, those 10 % of transcripts were only interrogated for NREs present in the 3’UTR region; however, Pum binds to the NRE located in the 3’ end of the open reading frame (ORF) in both *DmNa*_*v*_ and rat *Na*_*v*_ *1.6* [79, 78]. Therefore, there might be many more Pum targets yet to be identified.

In a genome-wide screening of transcripts associated with the RNA-binding region of Pum, more than 1,000 distinct mRNAs were identified [88]. This suggests that Pum is broadly involved in posttranscriptional regulation of many genes. Indeed, in addition to regulating translation of *Na*_*v*_s, Pum has also been implicated to regulate dendritogenesis [89, 90], expression of glutamate receptors [91], and aspects of memory and learning in higher brain centres [92]. Behaviour training of long-term memory (LTM) produced by spaced training (ten training sessions with a 15-min rest interval between each session), compared to anaesthesia-resistant memory (ARM) produced by massed training (ten training sessions without rest intervals), resulted in *pum* mRNA upregulation. Pum mutant flies also showed defects in LTM formation. [92]. Pum regulates NMJ morphology via negative regulation of the translational factor eIF-4E expression by directly binding to an NRE in the 3’UTR of the *eIF-4E* transcript [90]. Pum loss-of-function mutants show enhanced expression of eIF-4E and upregulated GluRIIA expression and increased frequency of spontaneous neurotransmitter release [91]. Thus, Pum is seemingly central to many aspects of CNS function, not least of which is homeostatic control of neuronal excitability. In this regard, it is significant that in mouse, Pum2 deficiency leads to spontaneous EEG abnormalities and lower seizure thresholds to the proconvulsant pentylenetetrazole [93]. Similar to Pum, the *Na*_*v*_ *1.6* transcript is upregulated in *CELF4* (CUGBP, ELAV-like family member 4) deficient mice [94]. CELF4 is similarly a brain-specific neuronal RNA-binding protein and binds to the 3’UTR of *Na*_*v*_ *1.6*. Because mammalian Na_v_1.6 is the primary determinant of action potential initiation and main contributor of I_NaP_ in excitatory neurons, upregulated *Na*_*v*_ *1.6* mRNA results in increasing neuronal excitability [95]. Consequently, *CELF4* deficient mice exhibit both convulsive and nonconvulsive (absence-like) seizures and also have a lower seizure threshold [94, 96]. These findings demonstrate that understanding the regulation of I_Na_ or I_NaP_ via RNA-binding proteins is a potentially important approach for epilepsy therapy.

### Summary and outlook

**Fig. 4:**
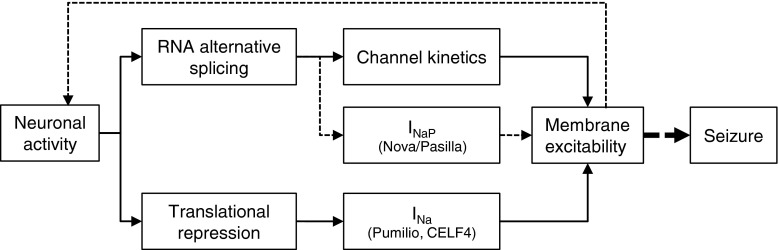
Membrane excitability is regulated by activity-dependent RNA alternative splicing and translational repression of voltage-gated sodium channel transcripts. Control of membrane excitability through Na_v_ activity is achieved by regulation of channel kinetics, current density (I_Na_), and magnitude of persistent Na current (I_NaP_). RNA alternative splicing results in splice variants which exhibit different channel gating properties including activation and inactivation kinetics and I_NaP_. Splicing is regulated, in part, by Pasilla in *Drosophila* and in humans by its homologue, Nova. In *Drosophila*, increased synaptic excitation results in increased I_NaP_, which in turn feeds back to further increase synaptic excitation. This self-reinforcing cycle likely further increases I_NaP_ (dashed line) leading to seizure. Current density of Na_v_ can be regulated through a mechanism of translational repression of *Na*_*v*_ transcripts via Pumilio and possibly CELF4

Voltage-gated sodium channels are important determinants for controlling membrane excitability. Regulation of Na_v_ activity is achieved, at least in part, by coordination of RNA alternative splicing and translational repression of *Na*_*v*_ transcripts (Fig. 4). When one considers additional mechanisms of regulation of Na_v_ channel activity, including RNA editing [28], phosphorylation [33, 32, 11, 4], trafficking [97, 98], and degradation [98–100], it becomes clear that these channels are subject to both considerable and diverse regulation consistent with the high level of channel diversity observable in the multitude of neuron types in the human brain. The utilisation of model systems, including *Drosophila*, offers the significant opportunity to rapidly progress understanding in these and related areas.

A particular area where *Drosophila* is already making a contribution to understanding epilepsy is through modelling human *Na*_*v*_ point mutations. A variety of techniques now exist to allow such mutations to be ‘knocked-in’ to *DmNa*_*v*_. Sun et al. [101] recently reported a *Na*_*v*_ *1.1* (K1270T) knock-in that recapitulates a mutation associated with genetic epilepsy with febrile seizures plus (GEFS+). Electrophysiological analysis shows this to be a gain-of-function mutation that results in a hyperpolarizing shift in the deactivation potential for I_NaP_. This approach not only serves to validate the genetic basis of human disease, but also provides a sensitised genetic background for high-throughput, low cost, screens to identify novel compounds that have antiepileptic properties. Identification of novel targets, such as splicing regulators, can also be quickly developed as the basis of screens with the potential advantage of identifying antiepileptic compounds which interact with nontraditional targets. By far the most common targets of currently used AEDs are ion channels and, whilst these offer effective therapeutic targets, there might be much to be gained from identifying additional targets which would facilitate combinatorial therapy. Combinations of AEDs are showing promise for the treatment of intractable epilepsy [102].

Use of *Drosophila* (and other simple model systems) also offers the prospect of exploring the mechanistic basis of epileptogenesis from understanding how small seizures may lead to larger seizures to providing novel approaches to prevent epilepsy from progressing, even when an epilepsy-associated mutation is present. For example, we recently reported that the presence of phenytoin, during embryogenesis when the CNS first forms neural circuits, prevents the normal seizure phenotype characteristic of the *Drosophila sda* mutant [48]. The inference from this study is that early intervention may be beneficial in blocking epileptogenesis by preventing activity-dependent feedback mechanisms that we spotlight in this review. The finding of conservation of regulatory mechanisms between insects such as *Drosophila* and mammals validates the use of simpler model organisms to provide better understanding of *Na*_*v*_ regulation in humans with an obvious benefit of novel therapies for epilepsy.
